# Tenir compte des origines dans le raisonnement médical en maladies infectieuses et tropicales ? Un regard critique

**DOI:** 10.48327/mtsi.v4i1.2024.362

**Published:** 2024-01-29

**Authors:** Amel FILALI, Lindsay OSEI, Nicolas VIGNIER

**Affiliations:** 1 Policlinique de médecine tropicale, voyages et vaccinations, Centre universitaire de médecine générale et santé publique, Lausanne, Suisse; 2 Service de pédiatrie, Centre hospitalier de Cayenne, Cayenne, Guyane, France; 3 Centre d'investigation clinique INSERM 1424, Centre hospitalier de Cayenne, Cayenne, Guyane, France; 4 Service des maladies infectieuses et tropicales, Hôpitaux universitaires Paris Seine-Saint-Denis, Hôpitaux Avicenne et Jean Verdier, AP-HP, Bobigny, France; 5 IAME (Infection, Antimicrobiens, Modélisation, Évolution), INSERM UMR 1137, Université de Paris, Université Sorbonne Paris Nord, Bobigny, France; 6 Institut Convergences Migrations, CNRS, Aubervilliers, France

**Keywords:** Migrants, Discrimination, Soins, Race, Maladies infectieuses, Formation médicale, Origine géographique, Déterminants sociaux, Ethnicité, Phénotype, Migrants, Discrimination, Healthcare, Race, Infectious diseases, Medical training, Geographical origin, Social determinants, Ethnicity, Phenotype

## Abstract

Les discriminations en soins liées aux origines font l'objet de plus en plus d'attention en médecine. La pandémie de Covid-19, par son ampleur, a contribué à leur mise en lumière. Des données, bien que peu nombreuses, existent en France sur leur existence. Ces discriminations ont un impact sur le parcours de soins et participent au renoncement aux soins des populations les plus concernées. La problématique des discriminations se pose de façon particulière pour les maladies infectieuses. En effet, l’épidémiologie des maladies infectieuses étant inégalement répartie dans le monde, des représentations sociales erronées sont fréquentes et exposent à un raccourci préjudiciable entre migrants et maladies infectieuses. Le caractère transmissible de certaines maladies infectieuses renforce leur potentiel de stigmatisation. Dans ce contexte, il est nécessaire de s'interroger sur la place qu'il convient de donner aux déterminants sociaux, à l'origine géographique, au phénotype et à l'ethnicité dans l'enseignement et le raisonnement médical. Le monde anglophone utilise le concept de « race » de manière structurelle sans que cette « norme internationale » n'ait, jusqu'alors, été appliquée en France dans une posture d'universalisme républicain. Afin d'améliorer la prise en soins des personnes faisant partie de groupes minoritaires, il apparaît important de mieux documenter et d'enseigner un raisonnement clinique basé sur l'origine qui soit plus nuancé, sans pour autant négliger l'importance du recueil et de la prise en compte des déterminants sociaux de la santé et des facteurs environnementaux.

## Abstract (en)

Healthcare discriminations based on one's ethnic background is increasingly being studied in medicine. The scale of the Covid-19 pandemic has played an important role in bringing them to light. Data, although scarce, exist in France. These discriminations have an impact on the care pathway and contribute to the renunciation of care by the most affected populations. The issue of discrimination is particularly relevant in infectious diseases. Although the epidemiology of infectious diseases is unevenly distributed worldwide, erroneous social representations are prevalent and expose to a harmful prejudice against migrants with regard to infectious diseases. The transmissible nature of some infectious diseases reinforces their stigmatizing potential. In this context, it seems important to discuss the dimension to be given to social determinants, geographical origin, phenotype, and ethnicity in teaching and medical reasoning. The English-speaking world uses the concept of “race” in a structural way, whereas this “international standard” has not been applied in France until now. To improve the care of people from minority groups, it seems important to better document and teach a more nuanced clinical reasoning based on origin, without neglecting the importance of collecting and taking into account social determinants of health and environmental factors.

## Introduction

L'exercice de la médecine s'inscrit dans des sociétés modelées par des courants idéologiques et est exposé à de fortes représentations sociales de la maladie. Loin d’être imperméable, la pensée médicale s'imprègne, de facto, des doctrines dominantes qui façonnent la société et son évolution. Ainsi, la médecine a contribué historiquement à l'extension du concept biologique de « race » des êtres humains ainsi qu’à leur hiérarchisation, et elle a servi de caution morale à l'entreprise coloniale des années qui ont suivi la révolution industrielle (xviii^e^-xix^e^ siècles) [38]. Arthur de Gobineau, dans son ouvrage *Essai sur l'inégalité des races humaines* (1853-1855), popularise le concept de « races » biologiques et promeut une vision racialiste, visant à diviser les êtres humains en différentes races qui selon lui pourraient être classées et hiérarchisées scientifiquement. Le pouvoir politique français de l’époque s'est également nourri de cette idéologie. Jules Ferry, premier ministre sous la III^e^ république, l'a reprise en ces termes dans son discours du 28 juillet 1885 : « Il faut dire ouvertement qu'en effet les races supérieures ont un droit vis-à-vis des races inférieures » [15]. Parallèlement, la littérature médicale a largement contribué à nourrir et valider scientifiquement les préjugés raciaux portant sur les populations colonisées (moindre perception de la douleur [21], infériorité intellectuelle [20], hypersexualité [4, 38]). Cela s'est notamment traduit par le développement de la craniométrie ou la mesure du quotient intellectuel [18, 20], basés sur le postulat que des différences existaient entre les « races », et ceci dans une finalité de hiérarchisation. Cette idée de différences entre les « races » est restée centrale dans le développement de certaines théories explicatives de différences observées entre groupes de populations et donc de discriminations, nonobstant le fait que d'autres facteurs, notamment socioéconomiques, environnementaux, culturels, géographiques puissent les expliquer.

Les discriminations ont encore aujourd'hui des conséquences importantes sur l'accès aux soins et le parcours de soins des populations faisant partie des groupes dits minoritaires (par opposition à la population dite majoritaire). Cette problématique se pose de façon singulière en infectiologie. La répartition inégale des maladies infectieuses dans le monde peut contribuer à alimenter des raccourcis préjudiciables entre migration et maladies infectieuses dans les représentations sociales. Dans ce contexte, et bien que la « race » soit un concept non reconnu en France, il est important de discuter de la place à accorder à l'origine géographique, au phénotype et à l'origine ethnique, mais aussi aux déterminants sociaux, dans l'enseignement et le raisonnement médical. L'objectif de cet article est de discuter de la place de ces différents critères et déterminants dans le raisonnement médical afin d'offrir une prise en charge la plus holistique et la moins biaisée possible.

## « Race » et médecine tropicale

Au sein de l'infectiologie, l'historique de la discipline qu'est la médecine tropicale est remarquable à ce titre car son développement est indissociable de la médecine coloniale [17, 36, 39]. Née au xix^e^ siècle avec pour objectif de prendre en charge la santé des personnes vivant dans les colonies, notamment en Afrique, continent qualifié alors de « tombeau de l'homme blanc » [10], et de lutter contre les « grandes endémies », elle s'inscrit dans le contexte de la colonisation européenne sur plusieurs continents. La colonisation a eu des effets majeurs sur la santé des peuples colonisés, du fait, outre le crime de l'esclavage, de l'introduction et de la diffusion de nouvelles maladies infectieuses avec comme conséquence une mortalité importante des populations naïves sur le plan immunologique. Ce fut en particulier le cas de la fièvre jaune en Amérique du Sud [19, 33]. Ainsi, améliorer la santé des colonisés s'intégrait également dans les missions initiales de la médecine tropicale et ce afin de faciliter la gestion des colonies. On peut citer à ce propos Patrick Manson, l'un des fondateurs de la discipline : « La médecine tropicale frappe, et frappe d'une manière efficace, à la racine même du problème principal de nos colonies – la maladie. La médecine tropicale va rendre moins onéreuse et plus efficace la gestion des colonies; elle va encourager et diminuer le coût du commerce, et elle améliorera nos relations avec les populations indigènes [31]. » Également dénommée pathologie « exotique » (du latin *exoticus* : étranger), il s'agit donc de la médecine qui s'occupe de l’étranger et du lointain. La Société francophone de médecine tropicale et santé internationale (SFMTSI), fondée en 1907, s'appelait encore Société de pathologie exotique jusqu'en 2019 [48]. Le terme même de médecine tropicale ou exotique est unique dans l'historiographie médicale. De fait, à quelques exceptions près (médecine générale, médecine interne, etc.), la dénomination des différentes spécialités médicales est définie par appareil ou par classe d’âge. La médecine tropicale/pathologie exotique est la seule qui soit définie par une aire géographique, la zone intertropicale en lien direct avec l'histoire coloniale et la lutte contre les grandes endémies [17].

## Sémantique et enjeux de l'emploi du terme « race »

La construction du terme « race » sur les plans historique, sociologique, politique ou même juridique diffère selon le pays dans lequel on se situe.

Aux États-Unis, le terme est usité de façon ancienne et sert de structure sur de nombreux plans, notamment sur le plan social, juridique ou politique. Dans leur ouvrage *Race in America,* Matthew Desmond et Mustafa Emirbayer précisent ainsi que la race est une « catégorie symbolique, basée sur le phénotype ou l'ascendance, construite dans des contextes sociaux et historiques particuliers et mal reconnue comme une catégorie naturelle » [13]. La notion de « race » est donc une construction sociale, et les catégories définies évoluent selon les époques.

En France, l'emploi de ce terme a ses spécificités historique et sociologique. L'approche prépondérante l'associe à un sens biologique historique ayant contribué, dans le contexte colonial, à justifier le racisme scientifique. La question raciale a ainsi doublement marqué l'histoire de France et l'histoire européenne (dans les colonies mais aussi dans l'Hexagone, notamment par l'antisémitisme). Nourri par cette histoire, un consensus politique antiraciste universaliste s'est construit, réunissant tout le champ politique français dans l'après-Seconde Guerre mondiale, sur fond de décolonisation. Utiliser le terme « race » est alors devenu une forme d'interdit moral. C'est « l'indifférence aux différences », selon la formule de Bourdieu, qui est supposée garantir l’égalité indépendamment des origines, qu'elles soient réelles ou attribuées. Ceci est annoncé en préambule de la Constitution de 1946 et repris dans l'article 2 de la Constitution de 1958 [9].

Aujourd'hui, les fondements du racisme ne sont plus aussi explicites, et la hiérarchie du biologique a cédé la place à la hiérarchie des cultures. Le mot ethnicité est ainsi parfois préféré au terme de race, et perçu par certains auteurs comme son euphémisme [9, 43]. En France comme ailleurs, alors que la notion de « race » n'a aucune validité en biologie humaine, elle reste pourtant présente dans les perceptions du sens commun. Le débat sur les discriminations a participé à réintroduire ces termes, et une terminologie soigneusement exclue du lexique post-1945 (en effaçant le terme « race » de la Constitution, le 12 juillet 2018 par exemple) refait surface. La mobilisation des catégories « ethno-raciales » dans la lutte contre les discriminations fait donc aujourd'hui controverse en France, tandis qu'elle constitue la grille de lecture principale de ces problématiques aux États-Unis, par exemple.

## Discriminations ethniques, sociales et accès aux soins

Les discriminations, qu'elles soient ressenties ou objectivées, sont de plus en plus étudiées dans le monde de la santé. La pandémie de Covid-19 a participé à mettre davantage en lumière ces discriminations structurelles qui sont une des causes des inégalités sociales de santé [25, 35, 43]. Aux États-Unis, où les statistiques ethniques ou phénotypiques sont largement répandues, les inégalités et les discriminations dans l'accès aux soins font l'objet d'une documentation de plus en plus importante. À titre d'exemple, une étude réalisée sur un échantillon de 172 549 enfants dans 186 centres médicaux à travers les États-Unis a montré un risque relatif de mortalité après une intervention chirurgicale de 3,43 (IC95% [1,73-6,79]) pour la population « noire » [35].

Bien que limitées en nombre et soumises à autorisation préalable, des statistiques en santé dites « ethniques » existent également en France, avec des données qui reposent plus souvent sur l'origine géographique (travaux sur les déterminants sociaux), et plus rarement sur l'apparence (travaux sur les discriminations). La production de statistiques en France est encadrée par la loi du 6 janvier 1978 relative à l'informatique, aux fichiers et aux libertés ainsi que par le Règlement général sur la protection des données (RGPD). L'article 6 de cette loi précise « qu'il est interdit de traiter des données à caractère personnel qui révèlent la prétendue origine ethnique ou raciale » [26]. L'article 9 du RGPD précise toutefois certaines exceptions. Le traitement de telles données est possible, notamment si elles s'avèrent nécessaires « à des fins archivistiques dans l'intérêt public, à des fins de recherche scientifique ou historique ou à des fins statistiques ».

Des données récentes existent donc, et seuls les travaux de recherche ayant fait l'objet d'une autorisation par les commissions *ad hoc* ont pu les recueillir.

Ces travaux ont notamment révélé que les femmes immigrées d'Afrique subsaharienne étaient plus susceptibles de recevoir des soins de qualité moindre dans le cadre de leur suivi de grossesse [40, 46]. Dans l'enquête « Trajectoires et origines » menée par l'Institut national d’études démographiques (INED) auprès d'un large échantillon représentatif de la population française, les personnes originaires d'Afrique subsaharienne déclaraient 3 fois plus souvent une expérience de discrimination dans leur accès aux soins [1]. Un autre travail mené sur les données de cette enquête objectivait des taux de discrimination significativement plus élevés pour les personnes appartenant à des groupes minoritaires ou défavorisés socialement : les femmes et les immigrés, en particulier ceux originaires d'Afrique subsaharienne et de Turquie, et les personnes nées en outre-mer [44]. Ces discriminations ont un impact sur le parcours de soins et participent au renoncement aux soins de ces populations. Avec une augmentation de 14 points de pourcentage de la probabilité de renoncer aux soins ultérieurs, les cas de non-recours aux soins apparaissent étroitement liés aux expériences de discrimination lors de soins passés. Ces discriminations ont également un effet à la fois sur la santé mentale et physique des personnes migrantes [34].

L’épidémiologie des maladies infectieuses varie selon les aires géographiques et parfois selon les groupes sociaux. Cette réalité sous-tend des représentations sociales souvent erronées et fréquentes qui exposent à un raccourci préjudiciable entre migrants, autres minorités visibles et maladies infectieuses. Ces représentations contribuent aux discriminations. Pour autant, l'inégale répartition mondiale des maladies infectieuses se traduit en effet par un risque accru de certaines maladies infectieuses chez les populations immigrées venant de zones à haute prévalence. Par exemple en France, les immigrés, qui sont définis comme toute personne née étrangère à l’étranger, représentent un tiers de l'ensemble des personnes vivant avec le VIH et près de la moitié des découvertes (dont un tiers sont acquises en France) [12]. Ils représentent 75 % des personnes vivant avec le VHB, 25 % des personnes vivant avec le VHC [12], et 84 % des paludismes d'importation [8, 24]. L'incidence de la tuberculose y est également 8 fois plus élevée en comparaison aux personnes nées en France. Des formes plus graves du fait du diagnostic tardif sont plus fréquemment rapportées, et une surmortalité par maladies infectieuses a également été démontrée à partir des données nationales [11,14,42,47,50]. De par leur nature potentiellement transmissible, les maladies infectieuses font l'objet de peurs et d'une stigmatisation importante. Les populations les plus concernées sont aussi souvent les plus socialement vulnérables. Les études montrent que ce sont souvent les facteurs géographiques et socio-économiques plutôt que l'origine ethnique qui expliquent la fréquence accrue de certaines maladies infectieuses [3].

L'enquête ANRS « PARCOURS » a démontré que la forte prévalence de l'infection à VIH chez les migrants d'Afrique subsaharienne n’était pas exclusivement liée au fait d’être originaire d'une zone de forte endémie, mais aussi à des contaminations fréquentes (35 à 49 %) sur le territoire français en lien avec des vulnérabilités sociales et sexuelles [51]. Ainsi, concernant l'infection à VIH, le fait de provenir d'une zone de haute prévalence est un facteur de risque a priori important; toutefois, ce facteur de risque ne doit pas occulter les conditions sociales qui déterminent autant le risque d'exposition. On se souviendra des débuts de l’épidémie d'infection par le VIH en France où 4 groupes furent identifiés et étiquetés comme « à risque » (les « 4H » : Haïtiens, hémophiles, héroïnomanes et homosexuels). Bien que traduisant en partie une réalité épidémiologique, cette catégorisation a contribué à la montée des discriminations vis-à-vis de ces groupes de population, qui persistent encore aujourd'hui. C'est pour en limiter l'impact que les autorités publiques ont par la suite contribué à l'invisibilisation des populations à risque, conduisant secondairement à une méconnaissance des enjeux de santé publique pour certaines populations clés. Ces éléments sont importants à prendre en compte, car ces données initialement produites pour une meilleure prise en charge des patients vulnérables peuvent être utilisées pour stigmatiser, dévoyant ainsi les enjeux de santé publique, comme en témoignent certaines dérives politico-médiatiques, notamment avec le concept d’« immigration bactérienne » mis en avant par Marine Le Pen en 2015 [32].

## Place des notions de « race », d'ethnicité et d'ascendance génétique dans le raisonnement diagnostic et la prise en soins des maladies infectieuses

À la lumière de ces données et de l'impact disproportionné de la Covid sur les populations vulnérables, un débat a lieu au sein de la communauté médicale et scientifique, notamment aux États-Unis sur le chemin à prendre pour lutter au mieux contre ces discriminations. La question de la place à accorder à la « race », à l'ethnicité ou à l'ascendance génétique dans le raisonnement clinique et la prise en soins des patients y occupe un rôle prépondérant. Le recours à ces termes étant sensible et la confusion souvent de mise, ceux-ci méritent d’être clarifiés quant à leur utilisation dans la littérature médicale. La « race » comme l'ethnicité sont des constructions sociales sans fondement biologique ou scientifique. Ces termes sont délicats à définir, car leurs définitions ont évolué et continueront à évoluer à mesure que les sociétés changent. Leur emploi dans la littérature médicale suscite par ailleurs de nombreuses réserves. L'ethnicité fait plutôt référence à l'histoire culturelle de la personne alors que la « race » correspondrait plutôt à une division de populations reposant sur des caractéristiques physiques [30]. Cette division varie en fonction des zones géographiques et des époques. Aux États-Unis, 5 catégories ont été utilisées pour le recensement effectué en 2000 : amérindienne, asiatique, noire ou afro-américaine, hawaïenne ou autre population océanienne, blanche. Une catégorie supplémentaire, hispanique, décrite comme « ethnicité » plutôt que « race » s'ajoute aux catégories indiquées précédemment [30]. Il est intéressant de noter que dans cette classification les Nord-Africains sont blancs, montrant à quel point ces catégories sont dessinées par le contexte historique et social de chaque pays. Au Brésil, les catégories sont au nombre de 5 mais différentes : blanche, noire, jaune, marron et indigène [22, 29]. Par ailleurs ces catégories ne sont pas figées et évoluent au fil du temps dans un même pays. De 1790 à 1850, les seules catégories existantes aux États-Unis étaient les populations blanche et noire.

L'ascendance génétique ferait plutôt référence à un pays ou une région d'origine. L'identification d'une ascendance génétique utilise les variations de fréquence allélique de points particulièrement variables de l'ADN, pour définir une ou plusieurs régions géographiques dont les ancêtres de l'individu étudié sont originaires. Il est toutefois important de noter que la majorité de la diversité génétique se situe au sein des populations et pas entre elles [18].

De telles définitions fondées sur des caractéristiques extérieures sont problématiques, imprécises et contribuent donc à nourrir les discriminations [16].

L'utilisation consciente ou inconsciente de ce vocabulaire a pour finalité, pour ses défenseurs, d'identifier les patients présentant un sur-risque de maladies (surtout pour les maladies avec susceptibilité génétique et les maladies infectieuses), d'orienter les choix thérapeutiques, ou d'utiliser des algorithmes diagnostiques corrigés par ces éléments.

La « race », construction socio-politique, révèle surtout, quand elle est utilisée, des disparités prenant racine dans les inégalités sociales et des discriminations subies, corrélées à une prévalence plus importante de certaines maladies. Ce n'est, à l'inverse, pas un bon marqueur de diversité génétique. Ainsi les Afro-Américains auraient approximativement 20 % d'ascendance européenne si on en croit les tests d'ascendance génétique [49]. Par ailleurs, la « race »/ethnicité attribuée par le personnel soignant n'est pas toujours le reflet de celle que les personnes concernées s'auto-attribuent. L’étude menée par Belbin *et al.* retrouvait en effet une discordance entre l'ethnicité/« race » attribuée dans le dossier médical et celle par laquelle le patient s'auto-définissait dans 35 % des cas [2]. Les résultats d'une étude menée par Lima-Costa *et al.* au Brésil sur 5 851 individus dans 3 régions distinctes du pays appuient également l'idée que l'autoclassification ethno-raciale est affectée à la fois par l'ascendance du génome mais aussi par des facteurs non biologiques. Par ailleurs, la force de l'association varie significativement entre les zones géographiques [29]. L'association entre l'auto-classification ethno-raciale et l'ascendance basée sur le génome n'est par ailleurs pas linéaire, les associations les plus cohérentes se situant aux extrémités de l’échelle de continuité de l'ascendance africaine.

L’étude de Bryc *et al.* qui a étudié l'ascendance génétique de 5 269 Afro-Américains, 8 663 Hispaniques et 148 789 Euro-Américains met quant à elle en lumière l'impact de siècles de mélange aux États-Unis [7]. Ceci montre la limite de ces regroupements d'individus en groupes distincts sans chevauchement dans des contextes biomédicaux qui ne peuvent pas être représentés de manière adéquate par des classifications. Leurs résultats démontrent notamment qu'au fil de centaines d'années, de nombreux individus avec une ascendance génétique africaine ou amérindienne se sont retrouvés dans la catégorie blanche.

Malgré ces limites, certains auteurs [6], mettant en avant une corrélation importante entre le continent d'origine ancestrale et la « race » auto-identifiée, s'opposent à l'abandon pur et simple de l'utilisation des catégories raciales ou ethniques [37]. Ils promeuvent une approche qui complèterait l'utilisation de la « race » et de l'ethnicité par des données relatives à l'ascendance génétique, les génotypes ou des biomarqueurs.

L'utilisation de l'ascendance génétique et/ou des catégories de populations comme alternative est une des propositions mise en avant dans la littérature anglo-saxonne récente, dans l'optique d'identifier des populations avec un risque génétique particulier. L'argument principal est que l'ascendance génétique permettrait un reflet plus fiable de la diversité génétique. Belbin *et al.* ont utilisé une méthode d'identité par descendance génétique qui permet d’évaluer l'ascendance commune en mesurant les segments d'ADN qui ont des séquences identiques : 96 % des participants de leur étude se retrouvent dans une des 17 communautés identifiées. Certaines de ces communautés (6 sur 17) étaient associées à un seul pays d'origine, tandis que d'autres dépassaient la « race »/ethnicité auto-déclarée [2]. Les détracteurs de cette démarche mettent en avant les limites de l'ascendance génétique dans la capacité à capturer la diversité génétique humaine. L'ascendance génétique, bien que perçue comme plus nuancée que la « race » ou l'ethnicité, est en fait un continuum y compris au sein des groupes tels que définis par la « race » et ou l'ethnicité. Par souci de précision, certains auteurs recommandent d'utiliser des termes spécifiques à l'origine géographique de l'individu, au-delà du continent ou du sous-continent, et d'insister sur la nécessité de tester les allèles à risque génétique plutôt que de présumer que les patients de certaines « races », « groupes ethniques » ou « populations » les portent par défaut [28]. Par ailleurs, il n'existe à ce jour pas de méthode validée et consensuelle pour établir cette ascendance génétique. Différentes méthodes existent aujourd'hui avec divers degrés de précision, de complexité et de coût (*identity by descent, local ancestry inference,* etc.).

Écarter totalement l'ascendance génétique du raisonnement médical se heurte à l'utilité de détecter des susceptibilités génétiques de maladies. Toutefois, les maladies les plus fréquemment identifiées lorsqu'on explore les différences entre populations en matière de santé, telles que le diabète et le cancer, ont des influences polygéniques et environnementales. Par ailleurs, les maladies monogéniques provoquées par une mutation ponctuelle unique qui a émergé sous une pression sélective sont une source moins courante de variation génétique chez les humains. Ces maladies monogéniques ont une distribution parfois hétérogène dans des populations, pouvant conférer des phénotypes spécifiques. Pour certaines maladies, ceci plaide de prime abord en faveur de l'utilisation du prisme de l'ascendance génétique dans la réflexion diagnostique [5]. L'exemple de la drépanocytose, provoquée par une mutation ponctuelle unique, est souvent cité. Toutefois, la répartition n'est de fait pas homogène dans les populations habituellement identifiées comme à risque, et réfléchir à l’échelle continentale n'est pas nécessairement pertinent (populations d'ascendance africaine notamment). Ainsi, notons pour la drépanocytose que la prévalence rapportée en Afrique du Sud et en Éthiopie est plus faible qu'en Espagne ou en France. L'Inde, par ailleurs, présente des prévalences plus élevées que la plupart des pays d'Afrique [41]. Cet exemple permet d'illustrer que les mutations influençant la couleur de peau ne permettent pas de prédire de façon fiable la présence d'autres mutations d'intérêt médical. Autrement dit, en dépit d'une ascendance génétique partagée, il n'y a pas nécessairement d'association préférentielle entre les différentes mutations étudiées dans ladite population; elles peuvent tout à fait fonctionner de manière indépendante d'un point de vue évolutif. Ces données sont donc intéressantes à l’échelle populationnelle, mais les traduire en outil dans le raisonnement clinique se heurte à l’écueil de la variance au sein de la population, avec un risque de porter préjudice à l'individu, si ces données étaient utilisées à visée diagnostique.

## Promouvoir l'utilisation des déterminants sociaux et culturels dans l'enseignement en médecine

À l'opposé d'une approche raciale, l'enseignement médical n'aborde pas suffisamment la complexité des déterminants sociaux à prendre en compte en soins courants. L'enseignement médical sur ces réalités est incomplet, et nécessite d’évoluer. La confusion entre « race », ethnicité, origine géographique, groupe culturel et religieux, statut administratif, et contexte socio-économique conduit à des raisonnements tronqués. Dans l’écrasante majorité des cas et, en particulier, en maladies infectieuses, recourir au prisme de l'ethnicité ou de l'ascendance génétique n'est ni pertinent, ni justifié et peut être préjudiciable. Le plus souvent, c'est l'origine géographique du patient qui est pertinente et son exposition avérée à des environnements et conditions de vie spécifiques et non son origine ethnique; il est pertinent ici de mentionner le cas des immigrés de deuxième et troisième générations qui n'ont souvent pas été exposés à l'environnement microbien du pays d'origine de leurs parents, mais restent identifiés comme venant d'ailleurs.

Ces derniers évoluent, à l'inverse, le plus souvent dans le même contexte socio-économique que leurs parents. Les conséquences des inégalités sociales sur leur santé peuvent donc être observées au sein de cette population. L'apprentissage de la médecine se fait par prototypes et par patients types. Cette démarche est justifiée dans l'enseignement tel qu'il est abordé, par une volonté d'identifier des groupes à risque, plus exposés à certaines pathologies, afin de prendre en compte les spécificités épidémiologiques de certaines maladies. Mais cela conduit régulièrement à des raccourcis parfois préjudiciables et à l'abandon d'une démarche nuancée. Ainsi, la tuberculose, par exemple, a certes une forte prévalence chez les primo-arrivants, mais voit son risque diminuer nettement au-delà de 10 ans de séjour, lorsqu'on s’éloigne d'un risque de contage dans le pays d'origine et que les conditions socio-économiques s'améliorent [45]. C'est donc le fait d'arriver d'un pays de forte incidence qui représente le principal facteur de risque et non l'ethnicité ou le pays de naissance seul.

## Conclusion

D'autres modèles de construction du raisonnement clinique sont proposés. Un individu malade ne peut être dissocié de son histoire de vie, de son histoire génétique, de la complexité de son environnement, de ses conditions de vie ou de ses interactions sociales. L'existence de vulnérabilités particulières peut dès lors être identifiée en tenant compte des déterminants sociaux et culturels de la santé plutôt que de celui de l'ethnicité. Cela semble d'autant plus important dans une discipline comme l'infectiologie où le pathogène, déclencheur de la maladie, est acquis.

Ces questions sont de plus en plus abordées dans la littérature médicale, notamment dans les pays anglo-saxons. Le moment apparaît propice pour qu'elles le soient davantage dans l'ensemble de l'Europe avec les spécificités liées à son histoire et à ses anciennes colonies. Ignorer ces problématiques ne règle en rien le problème des prises en charge de ces groupes minoritaires. Il apparaît donc raisonnable de prendre en compte ces éléments de manière plus approfondie pour dépasser l’« universalisme républicain » et s'orienter vers un universalisme proportionné. Cela passe par une meilleure documentation des discriminations dans l'accès aux soins en France, mais aussi par une modification du raisonnement épidémio-clinique en insistant sur le caractère bio-social des maladies infectieuses. Une meilleure compréhension des discriminations liées aux origines ethniques par le biais d’études rigoureuses est souhaitable, en utilisant le cadre règlementaire français qui autorise le recueil de ces origines ethniques et géographiques dans un contexte bien précis [23, 27].

Bien qu'un enseignement de l’éthique soit devenu la norme dans les facultés de médecine, l'enseignement des humanités médicales dans un sens plus englobant (histoire, philosophie de la médecine, anthropologie médicale) est insuffisant. Les soignants n'ont donc pas les clés suffisantes au cours de leur formation initiale ou continue pour la prise en compte de l'ensemble des déterminants sociaux et culturels de la santé dans la prise en soins de leurs patients. L'amélioration de l'enseignement prodigué aux futurs médecins sur ces déterminants, leur détection et la lutte contre les stéréotypes raciaux paraît à ce titre nécessaire.

## Remerciements

Nous remercions Loïc Epelboin et Michel Moutschen pour leurs remarques lors de la rédaction de cet article.

## Contribution des auteurs

AF, LO : conceptualisation, rédaction et correction du manuscrit.

NV : relecture et correction du manuscrit.

## Liens d'intérêt

Les auteurs ne déclarent aucun lien d'intérêt.
